# The Use of Patient-Reported Outcome Measures in Daily Clinical Practice of a Pediatric Nephrology Department

**DOI:** 10.3390/ijerph19095338

**Published:** 2022-04-27

**Authors:** Floor Veltkamp, Lorynn Teela, Hedy A. van Oers, Lotte Haverman, Antonia H. M. Bouts

**Affiliations:** 1 Department of Pediatric Nephrology, Emma Children’s Hospital, Amsterdam UMC, University of Amsterdam, Meibergdreef 9, 1105 AZ Amsterdam, The Netherlands; f.veltkamp@amsterdamumc.nl; 2 Child and Adolescent Psychiatry & Psychosocial Care, Amsterdam Reproduction and Development, Amsterdam Public Health, Emma Children’s Hospital, Amsterdam UMC, University of Amsterdam, Meibergdreef 9, 1105 AZ Amsterdam, The Netherlands; l.teela@amsterdamumc.nl (L.T.); h.a.vanoers@amsterdamumc.nl (H.A.v.O.); l.haverman@amsterdamumc.nl (L.H.)

**Keywords:** HRQoL, PROMs, chronic kidney disease, kidney transplantation, pediatric

## Abstract

(1) Background: Health-related quality of life (HRQoL) is lower in patients with chronic kidney disease (CKD) compared to the general population. In 2011, the KLIK PROM portal was implemented in the Emma Children’s Hospital to monitor and discuss HRQoL in daily care. This study describes and assesses the implementation and use of the KLIK PROM portal in the pediatric nephrology department. (2) Methods: CKD patients (self-report, if 8–18 years of age) and their parents (proxy-report, if 1–8 years) were invited to complete HRQoL patient-reported outcome measures (PROMs): TNO-AZL Preschool children Quality Of Life (TAPQOL) or Pediatric Quality of Life Inventory for Children (PedsQL). The PROMs were completed before and discussed during outpatient consultations. The adaptation rate—the proportion of patients/parents who were invited and completed at least one PROM—was calculated. Reported HRQoL scores of CKD patients were compared to the general population. (3) Results: In total, 142 patients (proxy- and self-report) were invited, 112 patients completed at least one PROM (adaptation rate 79%). Patients (*n* = 84 with informed consent for scientific use) with CKD reported lower HRQoL and HRQoL was more often impaired compared to the general Dutch population. (4) Conclusions: The implementation of KLIK was successful and its use is feasible for daily care. Using KLIK, HRQoL problems can be easily identified and monitored.

## 1. Introduction

Chronic Kidney Disease (CKD) in children is an irreversible and progressive disorder of any etiology, which has been present for more than three months [1]. The grade of CKD is determined by the kidney function, expressed as the estimated glomerular filtration rate (eGFR) [1]. Previous studies have shown that patients with CKD report a lower health-related quality of life (HRQoL) compared with the general population, irrespective of disease stage [2,3,4,5,6,7,8,9,10,11]. 

HRQoL is the perception of one’s position in life when put in terms of physical symptoms, functional status, and disease impact on psychological and social functioning [12,13]. To measure HRQoL, Patient Reported Outcome Measures (PROMs) can be used. PROMs are self- or proxy (parent)-reported questionnaires that measure the individual’s perception on the impact of disease and treatment in their life [14]. PROMs were initially developed for scientific research to study clinical outcomes and monitor disease progression and response to therapy. Nowadays, PROMs are more and more incorporated in daily clinical practice to facilitate the communication between clinician and patient/parent about outcomes important for patients, support shared-decision making, and increase satisfaction with provided health care [15,16]. Ultimately, this may improve the quality of health care and clinical outcomes, including survival rates [17,18,19]. 

**Figure 1:**
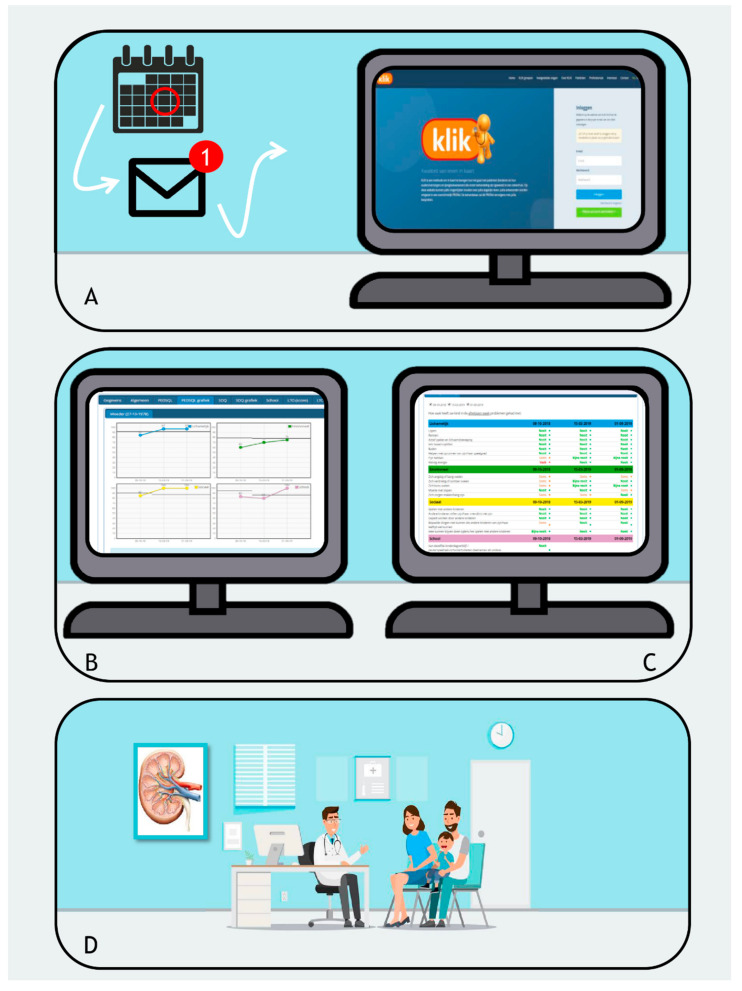
The KLIK method in daily clinical practice. (**A**) The patient and/or parents receive a reminder by email to complete the PROMs 7 days before a scheduled outpatient consultation. After completion, the answers of the PROMs are transformed into a KLIK ePROfile; (**B**) Literal representation of the answers using traffic light colors in order to easily identify items that need special attention (yellow or red); (**C**) HRQoL and change over time is visually displayed using graphs. The red line indicates reference data; (**D**) The clinician discusses the ePROfile with the patient and/or parents.

In 2011, the KLIK PROM portal (www.hetklikt.nu (accessed on 26 April 2022)) was implemented in the Emma Children’s Hospital, Amsterdam University Medical Center, a tertiary hospital in the Netherlands [20]. The main purpose of this portal is to use PROMs in daily clinical practice. The KLIK PROM portal contains validated PROMs regarding HRQoL, symptoms, parental distress, and/or psychosocial adaptation. Before outpatient consultation visits, patients and parents complete PROMs at home (Figure 1A). Answers of the PROMs are transformed into an electronic KLIK PROfile (ePROfile) that meets the wishes and preferences of clinicians and respondents [21,22,23,24]. Using a traffic light system for the literal answers (Figure 1B), problems can be easily identified (red = often problems, yellow = sometimes problems, and green = no problems), discussed, and be acted upon. PROM scores are shown in line graphs, so change over time is clearly visualized (Figure 1C). Additionally, reference data, corresponding with the average score for the general population, are shown in the graph as well. The clinician discusses the ePROfile with the patient and/or parents during the outpatient visits (Figure 1D). The KLIK PROM portal has been validated and its effectiveness has been previously described [20,25]. It is now implemented in over 70 different patient groups (e.g., rheumatology, oncology, and diabetes) in 25 hospitals in the Netherlands and 3 hospitals in the United Kingdom (www.klik-uk.org (accessed on 26 April 2022)) in both pediatric and adult healthcare. 

To demonstrate the usefulness and importance of the KLIK PROM portal to monitor HRQoL, we assessed its feasibility and conducted a retrospective, observational cohort study of the HRQoL data of patients followed at the pediatric nephrology department of the Emma Children’s Hospital. As one of the first patient groups, pediatric kidney patients have used KLIK since 2011.

## 2. Materials and Methods

### 2.1. The KLIK PROM Portal and Patient Selection

The KLIK PROM portal was implemented in the pediatric nephrology department of the Emma Children’s Hospital in July 2011. The department consists of three pediatric nephrologists and is one of the three pediatric kidney transplantation centers in the Netherlands. Before using KLIK in daily clinical practice, all clinicians were trained by the KLIK team in understanding and interpreting PROM scores using the ePROfiles [26]. 

Before the outpatient consultation, parents were invited by the pediatric nephrologist to register themselves and their child at the KLIK website (www.hetklikt.nu (accessed on 26 April 2022)). The pediatric nephrologist considered patients eligible if they suffered from a chronic kidney disease that required follow-up, including patients with a kidney transplant or patients on dialysis, if patients and/or their parents understood Dutch or English sufficiently, and if compliance was expected.

At registration, parents (until patient’s age of 16 years) and patients (12 years and older) indicate whether they consent with the use of their data for clinical practice and/or scientific purposes. Patients of 8 years and older complete the questionnaires by themselves (self-report). For children who are younger, parents complete the questionnaires on their behalf (proxy-report). A few days prior to the outpatient consultation, the KLIK expert team (a dedicated group of researchers with expertise in the field of PROMs and HRQOL research), checks if the patient registered and added the date of the next visit, and if so, the PROMs are made available automatically 7 days prior to their scheduled visit. Parents and/or patients receive a notification by email and, if not completed yet, a reminder followed by a phone call to complete the PROMs. Three months after the consultation, patients/parents receive a notification that asks them to add their next scheduled visit. A more detailed description of the KLIK PROM portal was published elsewhere [20,27].

### 2.2. Feasibility

To assess feasibility of the KLIK PROM portal in the pediatric nephrology department, the following feasibility parameters were used: the proportion of patients/parents who (1) registered after invitation (invited before 1 February 2020), (2) completed at least 1 PROM before 1 August 2020, and (3) reported discussion of the ePROfile with the clinician. All patients who received an invitation to KLIK are reported to the KLIK team. To check if the ePROfile was discussed during consultation, the electronic patient file of each patient was screened for the terms “KLIK”, “quality of life”, or “questionnaire(s)”.

If the adaptation rate, defined as the proportion of invited patients that completed at least one questionnaire (parameter number 2 divided by total invitations), was ≥70%, the KLIK PROM portal was considered feasible. Additionally, each of the three feasibility parameters should exceed 70%. The cut-off value of 70% was based on a previous study [20].

### 2.3. Measures

Patients of the pediatric nephrology department were asked to complete both generic and disease-specific questionnaires in the KLIK PROM portal as part of standard care. To measure HRQoL of CKD patients, two different generic PROMs were used: the TNO-AZL Preschool children Quality Of Life (TAPQOL) for children under the age of 5, and the Pediatric Quality of Life Inventory (PedsQL) Generic Scale 4.0 for children aged 2–4 years, 5–7 years, 8–12 years, and 13–18 years old. Only the generic PROMs were used for the analysis. A complete overview of all PROMs that are currently used in daily clinical practice at the pediatric nephrology department can be found in the Supplementary Table S1 online.

#### 2.3.1. TNO-AZL Preschool Children Quality of Life (TAPQOL)

The TAPQOL questionnaire was used to measure HRQoL of children 0–5 years of age. It consists of 43 items divided over 12 scales: sleeping, appetite, lung problems, stomach problems, skin problems, motor functioning, problem behavior, social functioning, cognitive functioning, positive mood, anxiety, and liveliness. Scores are linearly transformed into a 0–100 score per scale, with higher scores indicating a better HRQoL. Questionnaires are completed by parents only. The TAPQOL has good validity and psychometric performance and reference data from the general population are available and used as reference group. A pooled mean and standard deviation (SD) of the general population were calculated using the mean ± SD scores of healthy, preterm, and chronically ill children combined [28]. In 2018, the TAPQOL was replaced by the PedsQL 4.0 for 2–4 years, but is still used for 0–1-year-olds.

#### 2.3.2. Pediatric Quality of Life Inventory (PedsQL) Generic Scale 4.0

The PedsQL was used to measure HRQoL of children between 2 and 18 years of age. It includes both proxy- (2–7 years) and self- (8–18 years) report. The proxy (5–7 years) and self-report versions (8–18 years) contain 23 items in four subscales: physical (8 items), emotional (5), social (5), and school functioning (5) [29]. The 2–4 years version includes 21 items (only 3 school/nursery functioning items). A 5-point Likert scale is used to indicate to what extent the child has difficulties with a certain problem (1 = never a problem, 2 = almost never a problem, 3 = sometimes a problem, 4 = often a problem, 5 = almost always a problem). Items are reverse-scored and transformed to a 0–100 scale (1 = 100, 2 = 75, 3 = 50, 4 = 25, 5 = 0). Higher scores indicate better HRQoL. The PedsQL has a short completion time, and has good feasibility, validity, and reliability [29,30]. There was high internal consistency reliability of the total score and subscales for all age groups. Additionally, reference data of representative and large samples of the Dutch population are available and used as reference group [31,32].

### 2.4. Statistical Analysis

Baseline patient characteristics (age and sex) were collected from the electronic patient file and are presented as means ± SD or medians (range), according to distribution, or as proportions (%). Baseline differences between groups (i.e., responders vs. non-responders and patients vs. reference group) in age and sex were tested using a Student’s *t*-test and Fisher’s exact test or Mann–Whitney U test, depending on distribution, respectively. Additionally, the total number of generic PROMs completed by each patient was collected. 

To compare the HRQoL of CKD patients with the reference group, only data from the first completed PROM from patients/parents who gave informed consent to use their data for scientific purposes at registration were used. The difference in mean subscale scores of the TAPQOL questionnaire and the total and subscale PedsQL scores was calculated using a one and two sample Student’s *t*-tests, respectively. The proportion of patients with an impaired overall HRQoL and HRQoL per subscale was based on a PedsQL score of more than 1 SD below the mean of the reference group [7,10]. By definition, 16% of the general population reports impaired HRQoL. A *p*-value of <0.05 was considered significant. 

Data analyses were performed using IBM SPSS Statistics for Windows (IBM Corp. Released 2017. Version 25.0. Armonk, NY, USA: IBM Corp.) for HRQoL scores (PedsQL and TAPQOL), and R Studio (version 3.6.1, 2019, Vienna, Austria. https://r-project.org (accessed on 18 April 2022)) for the realization of the graphs [33].

### 2.5. Ethical Considerations

This retrospective study was considered not being subject to the Medical Research Involving Human Subjects Act (WMO) by the Medical Ethical Committee of the Amsterdam UMC, location AMC (decision #W19_125). The study was approved by and a waiver of informed consent was obtained from the Medical Ethical Committee of the Amsterdam UMC, location AMC. However, for the purpose of the HRQoL analysis, patients (≥12 years of age) and/or their legal guardians need to give their informed consent to use their data for scientific research. To use the data of patients from the KLIK PROM portal in daily clinical care, informed consent from patients and parents is mandatory. All research was performed in accordance with the relevant guidelines and regulations.

## 3. Results

The mean ± SD age of the patients who were invited was 10.0 ± 4.7 years; more boys (58%) than girls were invited.

### 3.1. Feasibility

**Figure 2:**
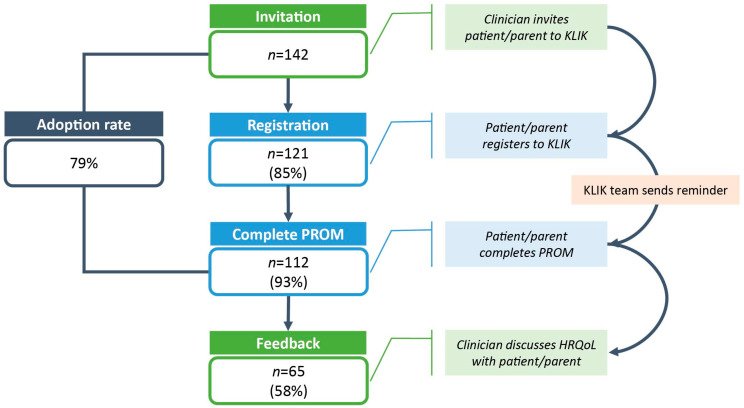
Flowchart of from the implementation of the KLIK PROM portal in the pediatric nephrology department. The adaptation rate is the percentage of patients/parents who were invited to the KLIK PROM portal and completed at least one PROM.

Between July 2011 and February 2020, a total of 142 patients were invited by letter for registration by the clinician, of which 121 (85%) registered themselves on the KLIK PROM portal. Of the registered patients, 112 (92%) completed at least one PROM. Feedback from the clinician—as documented in the electronic patient file—was received by 65 patients (58%). Of all the parents/patients who were invited, 79% completed at least one PROM (adaptation rate) (Figure 2). Patients who did not register or did not complete a PROM, tended to be older than patients who did, but this was not significant (*p* = 0.09 and *p* = 0.06, respectively). No difference in sex distribution was present (Table 1). The mean ± SD age at which the first PROM was completed was 10.1 ± 4.6 years. There was a wide range in the frequency of completing PROMs before an outpatient visit since registration on the KLIK portal (range 1 to 30, median 7) as well as a wide range in the number of years active on the portal (range 0 to 8.9 years, median 3.0 years). Sixteen patients completed PROMs on only one occasion.

### 3.2. HRQoL Differences

Of those who completed at least one PROM, 86 (77%) patients and/or parents gave informed consent for the use of their data in scientific research. Table 2 and Table 3 display the age and sex distribution of the CKD and reference group for each PROM category: (a) TAPQOL, (b) PedsQL proxy-report (5–7 years), and (c) PedsQL self-report (8–18 years). For TAPQOL, the age ranged between 10 to 60 months in the reference group and between 4 and 66 months in CKD patients. For PedsQL, no differences were found in sex and mean age between the CKD and reference group.

CKD patients aged 0–5 years (*n* = 22) scored significantly lower on 3 of the 12 TAPQOL subscales compared to the reference group: appetite (*p* = 0.02), stomach problems (*p* = 0.001), and motor functioning (*p* = 0.03) (Table 2). The mean score for problem behavior was higher in CKD patients (*p* = 0.03). For CKD patients aged 5–7 years (n = 10), no differences in HRQoL compared to the reference group were found, except on physical functioning (*p* = 0.038) (Table 3). For children aged 8–18 years (n = 54), CKD patients scored significantly lower on total PedsQL score (*p* = 0.001), and on the subscales physical functioning (*p* < 0.001), school functioning (*p* = 0.002), and psychosocial functioning (*p* = 0.033) (Table 3). 

**Figure 3:**
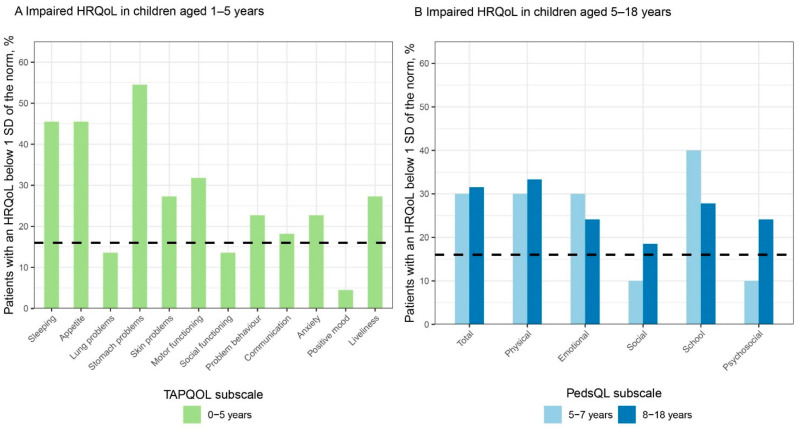
Bar plot of the proportion (%) of impaired (**A**) TAPQOL subscale scores (*n* = 22) and (**B**) total PedsQL score and subscale scores for proxy-report (5–7 years, *n* = 10) and self-report (8–18 years, *n* = 54) in CKD patients. Impaired HRQoL is defined as a score below 1 SD of the mean score of the reference group. By definition, the prevalence of impaired HRQoL in the reference group is 16% for each domain (represented by the horizontal dashed line).

Impaired HRQoL was reported in CKD patients in all but three domains of the TAPQOL questionnaire (lung problems, social functioning, and positive mood) (Figure 3A). Overall HRQoL was impaired in 30% (n = 3) and 31.5% (*n* = 17) of the CKD patients who completed the PedsQL 5–7 years and PedsQL 8–18 years, respectively. In the 5–7-year olds, school functioning was most often impaired (40%), whereas physical functioning was most often impaired in the 8–18-year olds (33%) (Figure 3B).

**Table 1:** Demographics of subjects who were invited to use the KLIK PROM portal. Demographics were compared between CKD patients who registered at the KLIK PROM portal and who did not, and of those, who completed at least one PROM and who did not. CKD = chronic kidney disease; IQR = interquartile range; and SD = standard deviation.

	Registered	Not Registered	*p*-Value
***Registration, n***	121	21	
Age at invitation (years), mean ± SD	9.7 ± 4.8	11.5 ± 4.3	0.09 ^1^
Male, *n* (%)	68 (56)	14 (67)	0.48 ^2^
	**≥1 PROM Completed**	**No PROM Completed**	
***Completion, n***	112	9	
Age at registration (years), median (IQR)	10.2 (6.1–13.4)	15.6 (9.0–16.3)	0.06 ^3^
Male, *n* (%)	64 (57)	4 (44)	0.50 ^2^

^1^ By Student’s *t*-test. ^2^ By Fisher’s exact test. ^3^ By Mann–Whitney U-test.

**Table 2:** Scores per subdomain of the TAPQOL. Scores are presented for CKD patient and reference group (general population) aged 0–5 years (proxy-report). Significant differences are shown in bold. CKD = chronic kidney disease; NA = not available; and SD = standard deviation.

	CKD 0–5 y (*n* = 22)	Reference 1–5 y (*n* = 378)	*p*-Value
Age (months), range	4–66	10–60	*-*
Male, *n* (%)	14 (63.6)	*NA* ^2^	*-*
Sleeping	68.2 ± 31.6	80.9 ± 18.1	0.07
Appetite	70.8 ± 25.2	84.3 ± 13.7	**0.02**
Lung problems	92.4 ± 18.3	92.0 ± 15.8	0.92
Stomach problems	75.8 ± 18.0	90.3 ± 15.3	**0.001**
Skin problems	83.0 ± 24.7	91.6 ± 11.5	0.12
Motor functioning ^1^	88.5 ± 17.5	97.2 ± 6.6	**0.03**
Social functioning ^1^	90.4 ± 17.0	89.8 ± 16.9	0.89
Problem behavior	76.6 ± 20.0	66.9 ± 16.3	**0.03**
Communication ^1^	83.2 ± 26.1	89.9 ± 11.2	0.24
Anxiety	76.5 ± 19.0	76.5 ± 18.3	>0.99
Positive mood	96.2 ± 17.8	98.6 ± 6.6	0.53
Liveliness	90.9 ± 16.0	97.6 ± 9.1	0.06

^1^ Only asked to children aged 18 months and older. Question was answered by 19 and 58 subjects of the CKD and reference group, respectively. ^2^ No information on sex distribution for all age groups combined was available.

**Table 3:** Total scores and scores per subdomain of the PedsQL Generic 4.0. Scores (mean ± SD) are presented for CKD patients and the reference (general population) group aged 5–7 years (proxy-report) and aged 8–18 years (self-report). Psychosocial functioning consists of the emotional, social, and school functioning subdomains. Significant differences are shown in bold. CKD = chronic kidney disease; SD = standard deviation.

	CKD 5–7 (*n* = 10)	Reference 5–7 (*n* = 274)	*p*-Value	CKD 8–18 (*n* = 54)	Reference 8–18 (*n* = 1012)	*p*-Value
Age (years), mean ± SD	7.2 ± 0.6	6.5 ± 0.9	0.076	12.8 ± 3.1	13.4 ± 3.0	0.557
Male, *n* (%)	4 (40)	152 (55.5)	0.33	33 (61.1)	521 (51.5)	0.17
Total score	79.1 ± 12.2	86.0 ± 11.6	0.065	79.1 ± 12.1	84.9 ± 12.6	**0.001**
Physical functioning	82.5 ± 15.1	91.1 ± 12.6	0.038	83.0 ± 14.9	91.5 ± 12.4	**<0.001**
Emotional functioning	70.5 ± 4.3	77.9 ± 16.5	0.162	75.4 ± 17.9	79.2 ± 18.3	0.133
Social functioning	85.5 ± 13.8	86.4 ± 16.7	0.868	82.7 ± 17.0	84.5 ± 16.9	0.451
School functioning	76.0 ± 20.1	85.8 ± 15.4	0.051	73.1 ± 16.9	80.5 ± 16.7	**0.002**
Psychosocial functioning	77.3 ± 13.5	83.4 ± 13.7	0.170	77.0 ± 12.6	81.4 ± 14.6	**0.033**

## 4. Discussion

This study shows that the use of the KLIK PROM platform in daily clinical practice of a pediatric nephrology department is feasible with an adaptation rate of 79%, i.e., the number of patients and/or their parents who were invited and completed at least one PROM. Except for (documented) discussion of the ePROfile by the clinician, the feasibility parameters met the 70% cut-off value. The value of using PROMs in daily clinical care is twofold: on an individual and group level. Individual scores are presented graphically and can be easily interpreted. In this way, impaired HRQoL can be captured by the KLIK PROM portal, which may allow for a timely intervention. On a group level, the value of KLIK is further supported by the analysis of HRQOL in 2–18 year-olds. The results of this study confirm that not only in clinical trials, but also in daily clinical care, patients with CKD report lower HRQoL and that HRQoL is more often impaired compared to the reference group. 

According to the Consolidated Framework for Implementation Research (CFIR), which comprises five domains ((1) intervention characteristics, (2) inner and (3) outer setting, (4) characteristics of individuals, and (5) process by which implementation is accomplished) [34], several factors have positively contributed to the feasibility of the KLIK PROM portal in general. The Emma Children’s Hospital provided the environment and promoted the use of the KLIK PROM portal (inner setting) and clinicians were trained on how to use PROMs in daily clinical care (inner setting and characteristics of individuals). The team of clinicians was in turn motivated by a local champion who actively promotes the implementation (process of implementation). The compliance of patients and/parents is enhanced by the short completion time of the PROMs, feedback by clear and direct visualization of their answers over time, and a user-friendly environment (intervention characteristics). A recent survey reports that clinicians are satisfied with the KLIK PROM portal [35,36]. Furthermore, it is known that patients and parents valued the use of the KLIK PROM portal as the results are also discussed with the patients, giving them insight into their functioning, helping them preparing for the consultation, and resulting in a broader range of topics to discuss with the clinician [37]. To improve feedback rates and user-friendliness, a front-end integration of the KLIK portal into the electronic patient file was successfully completed recently [35]. Additionally, KLIK is now also available as a web application for mobile devices. 

The use of PROMs in clinical care is potentially hindered by some practical barriers that might challenge feasibility. This was also partly exhibited in this department. An important limitation to this study is that patients were subjectively selected by the clinician and asked for participation (characteristics of individuals). Patients with poor compliance, limited resources, complicated housing situations, or other reasons were often not invited or declined the invitation. A significant proportion of the patient population consists of patients with a language barrier, who were not considered for participation. This may have biased the results, as previous research has shown that these groups are especially prone to (psychosocial) problems and report lower HRQoL [38,39]. Additionally, this is a missed opportunity: they could greatly benefit from closer monitoring by the use of ePROfiles. Second, the ePROfile was discussed by the clinician at least once with only 58% of the patients (characteristics of individuals). Although only documented discussion could be used for this study—therefore, this proportion may be an underestimation—there is ample room for improvement. In a recent KLIK evaluation study, patients (52%) and parents (45%) mentioned a similar feedback rate [37], whereas 70% of the clinicians report that they (almost) always discuss the ePROfile with the patient [35]. Discussing PROMs by the clinician is an important incentive for patients to use the KLIK PROM portal [36]. Besides implementation of the front-end integration in the electronic patient file, actively encouraging patients to bring up PROs by providing educational videos and topic lists, an improved training program for clinicians focused on the discussion of PROMs, and appointing a local champion to promote PROM discussion are also used to increase feedback rates. Third, compliance to completing the questionnaires differs between patients as is shown by the wide range in the frequency of completing PROMs before consultation (characteristics of individuals). However, this may be biased by the number of visits and how recently the patient was invited. The KLIK expert team aims to increase the compliance by contacting patients and/or their parents before a scheduled outpatient visit. This personal approach by a local dedicated team contributes to the high completion rate and is—despite a high work load—highly recommended for successful PROM implementation. 

Electronically collected individual PRO data support the clinician in person-centered care and the patient in engagement more effectively, ultimately leading to improved satisfaction, health, and HRQoL outcomes, while aggregated PRO data that is publicly available may create pressure from the patients to improve the quality of care, allow for comparison with individual scores, and use in shared decision making. A recently published realist synthesis reviewed the theoretical and empirical data that support the different uses of PROs [40]. The KLIK PROM portal aims to follow these uses: individual scores are discussed to guide the clinician in daily care and help the patient/parent to engage during the visit. The comparison of individual scores with reference data facilitate the interpretation as well as identifying potential problems. 

In this study, the data was not collected within a structured research setting. This may limit the interpretation of the results. Only the first completed PROM was used, which may have been at different stages in the disease course for an individual patient. However, the results show that—also in daily clinical practice—CKD patients report lower HRQoL scores. Furthermore, sample sizes of the CKD groups were small. Differences in socio-demographic background characteristics (family situation, education, occupation, and mental health status) between the CKD group and the reference group could have biased the results.

**Figure 4:**
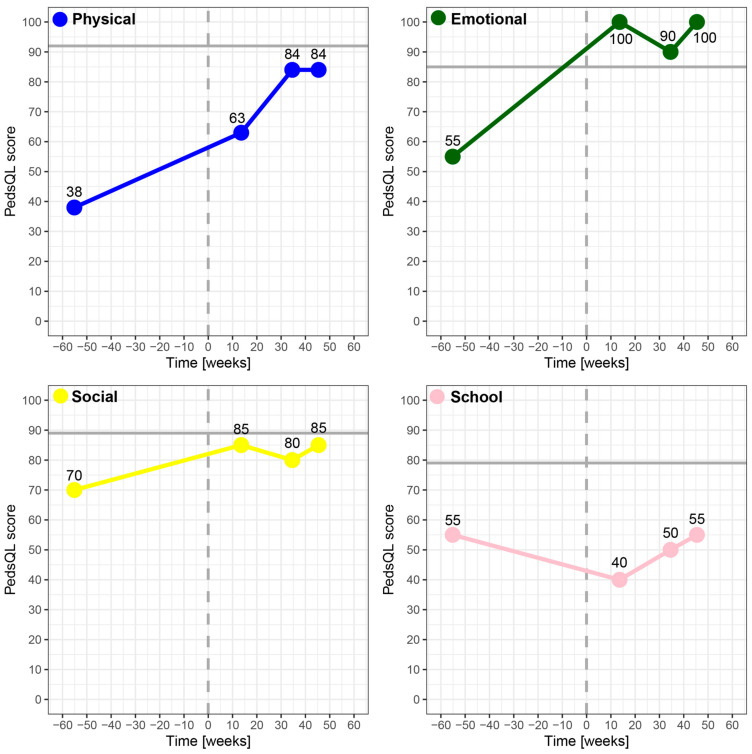
Visual graph of a kidney transplant patient whose overall HRQoL, measured with the PedsQL, improved following transplantation (vertical dashed line), but would still be below the overall mean score of the reference group (horizontal solid line). Before transplantation, this patient was on hemodialysis after its renal function worsened progressively within a year. The patient experienced extreme fatigue and found it difficult to cope with its diet restrictions. As a result of the progressive course of the disease, the patient had trouble sleeping for which they were referred to psychosocial care. Several weeks after successful transplantation, the patient had to be admitted to the hospital with a urinary tract infection, which was adequately treated. There were no signs of rejection. In the months following transplantation, the arterial-venous shunt in the patient’s arm was surgically removed upon own request, which may have led to a further improvement of the HRQoL scores.

Following the ISOQOL user’s guide for implementing PROMs in daily clinical practice [41], several choices can be made. These include the choice of which patient group, which PROMs, frequency and administration of PROMs, and tools for interpretation and presentation of the results. In our patient population, next to generic PROMs measuring HRQoL, disease-specific PROMS (e.g., PedsQL Transplant Module 3.0 for solid organ transplant recipients) [42] and PROMs related to medication use, school, and parental distress are also used in the nephrology department with the use of KLIK [20]. As a result, the clinician has a complete overview of the patient’s and parent’s wellbeing when looking at the ePROfile. To facilitate presentation and interpretation of the results, the traffic light system makes it easy to recognize individual items as potential hazards to the HRQoL, whereas the graphical display of the summarized scores shows the change over time and how it compares to the reference group (Figure 4: Change in HRQoL following kidney transplantation). With these choices, the goal of the KLIK PROM portal—to easily identify impaired HRQoL and psychosocial and/or physical problems to facilitate timely and adequate intervention—can be achieved. Although it was not the aim of the present study and no data on this outcome has been collected, in a previous study by the KLIK team, it was shown that users of the portal from multiple departments were—although not significantly—more often referred to another specialist than non-users [25]. 

## 5. Conclusions

This study shows that the implementation of the online KLIK PROM portal was feasible. Our results showed that the KLIK PROM portal is able to capture lower HRQoL in CKD patients by collecting PROMs in daily clinical practice. Monitoring HRQoL in daily practice on an individual level could positively contribute to evaluate the effect of the treatment but also to shared decision making and patient/parent satisfaction with provided healthcare of patients with any kind of chronic illness or condition. 

## Data Availability

The datasets generated and/or analyzed during the current study are available from the corresponding author upon reasonable request.

## Supplementary Materials

The following supporting information can be downloaded at: https://www.mdpi.com/article/10.3390/ijerph19095338/s1, Table S1: Overview of all PROMs that are currently used for the Chronic Kidney Disease group and the Kidney Transplantation group at the pediatric nephrology department of the Emma Children’s Hospital.
