# Supplementary material for: The Use of Patient-Reported Outcome Measures in Daily Clinical Practice of a Pediatric Nephrology Department
Source: Int J Environ Res Public Health. 2022 Apr 27;19(9):5338. doi: 10.3390/ijerph19095338 (PMC9102002; doi:10.3390/ijerph19095338)
Supplement: Supplementary file 1 [file ijerph-19-05338-s001.zip › ijerph-1692907-supplementary.pdf]

Supplementary Files

International Journal of Environmental Health and Public Research

Supplement to:

The use of Patient-Reported Outcome Measures in daily clinical practice of a pediatric nephrology department.

Floor Veltkamp, Lorynn Teela, Hedy A. van Oers, Lotte Haverman, Antonia H.M. Bouts.

Corresponding author: Antonia H.M. Bouts, Emma Children's Hospital, Amsterdam UMC, University of Amsterdam, Department of Pediatric Nephrology, Meibergdreef 9, 1105 AZ Amsterdam, the Netherlands; a.h.bouts@amsterdamumc.nl.

Table S1: Overview of all PROMs that are currently used for the Chronic Kidney Disease group and the Kidney Transplantation group at the pediatric nephrology department of the Emma Children's Hospital.

	Proxy-report		Self-report			
**Age range**	**0-1 year**	**2-7 years**	**8-11 years**	**12-15 years**	**16-18 years**	**Frequency**
**Informed consent**	Parent	Parent	Parent	Parent + Child	Child	Once
**PROM**	**Parent about self**		**Parent about self**			
**Sociodemographic**	Sociodemographic		Sociodemographic			1x per 2 years
**Symptoms of distress**	DT-P		DT-P			Once a year
**PROM**	**Parent about child**		**Child about self**			
**HRQoL child**	TAPQOL	PedsQL / TAPQOL*	PedsQL			Each visit
**HRQoL (Transplantation)**	*NA*	PedsQL Transplant	PedsQL Transplant			Each visit
**Skill for growing up**	*NA*					Once a year
**Problems with medication**	Problems with medication		Problems with medication			Each visit
**Therapy compliance**	Therapy compliance		Therapy compliance			Each visit
**Medication & appearance**	*NA*		Medication & appearance			Each visit
**School**	*NA*		School			Each visit

* In 2018, the TAPQOL was replaced by the PedQL 4.0 (2-4) and PedsQL (5-7) for patients aged 2-5 years.

For each age group using the PedsQL questionnaires, age-specific PROMs are provided (PedsQL 2-4 years, PedsQL 5-7 years, PedsQL 8-12 years, and PedsQL 13-18 years). PROMs measuring disease-specific HRQoL concerning transplantation are only completed by patients who underwent a kidney transplant. The PROM measuring problems with medication is only completed by the Chronic Kidney Disease group. HRQoL = Health-related quality of life; NA = Not applicable; DT-P = Distress Thermometer for Parents, PedsQL = Pediatric Quality of Life Generic Inventory 4.0; PROM = Patient reported outcome measure; TAPQOL = TNO-AZL Preschool children Quality Of Life.
